# Supplementary material for: Seminoma and Embryonal Carcinoma Footprints Identified by Analysis of Integrated Genome-Wide Epigenetic and Expression Profiles of Germ Cell Cancer Cell Lines
Source: PLoS One. 2014 Jun 2;9(6):e98330. doi: 10.1371/journal.pone.0098330 (PMC4041891; doi:10.1371/journal.pone.0098330)
Supplement: File S1 — ZIP file containing DMRforPairs output for significant regions. Please start from the html files. (ZIP) [file pone.0098330.s008.zip › figures/22309.pdf]

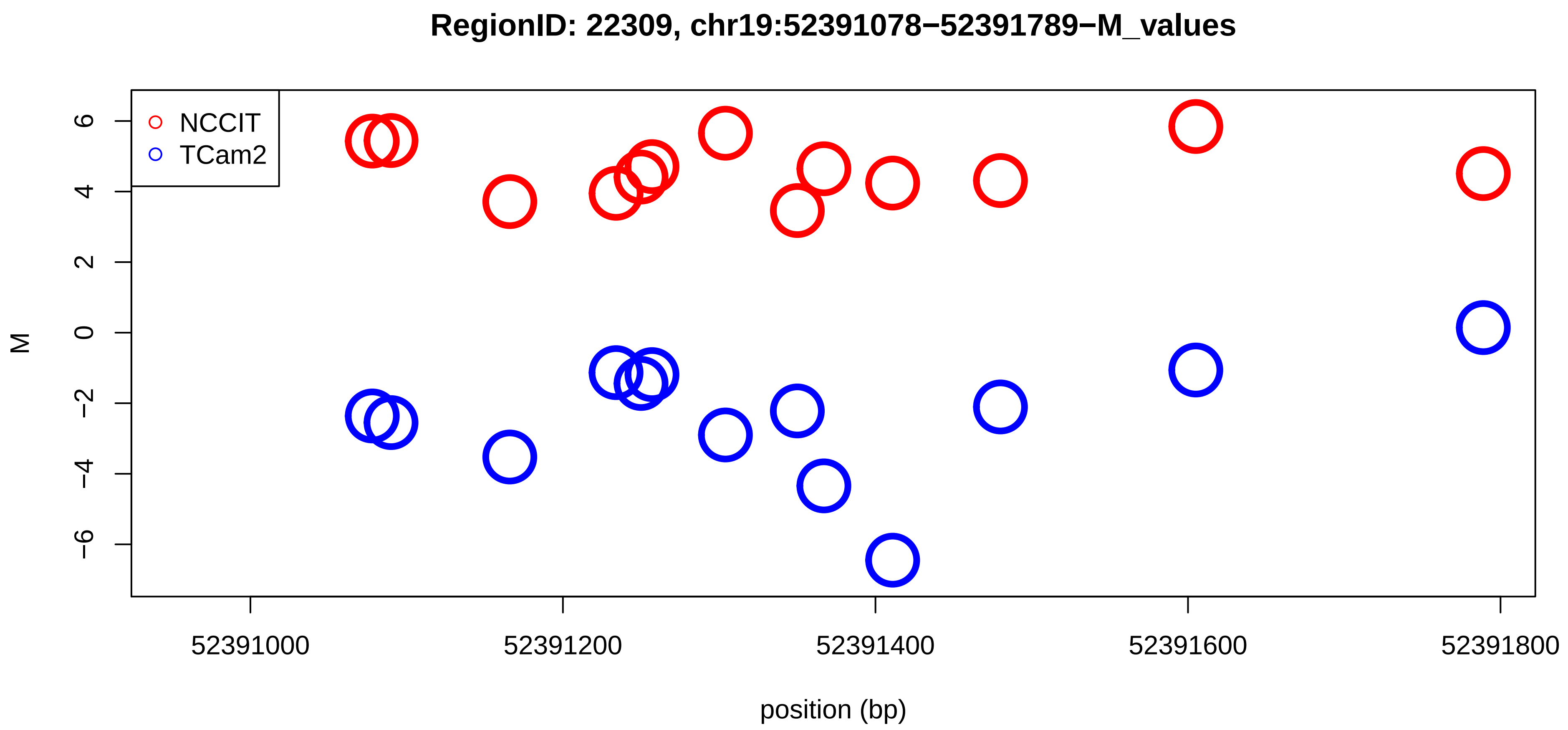
RegionID: 22309, chr19:52391078−52391789−M_values
NCCIT
TCam2
M
6
4
2
0
−2
−4
−6
52391000
52391200
52391400
52391600
52391800
position (bp)

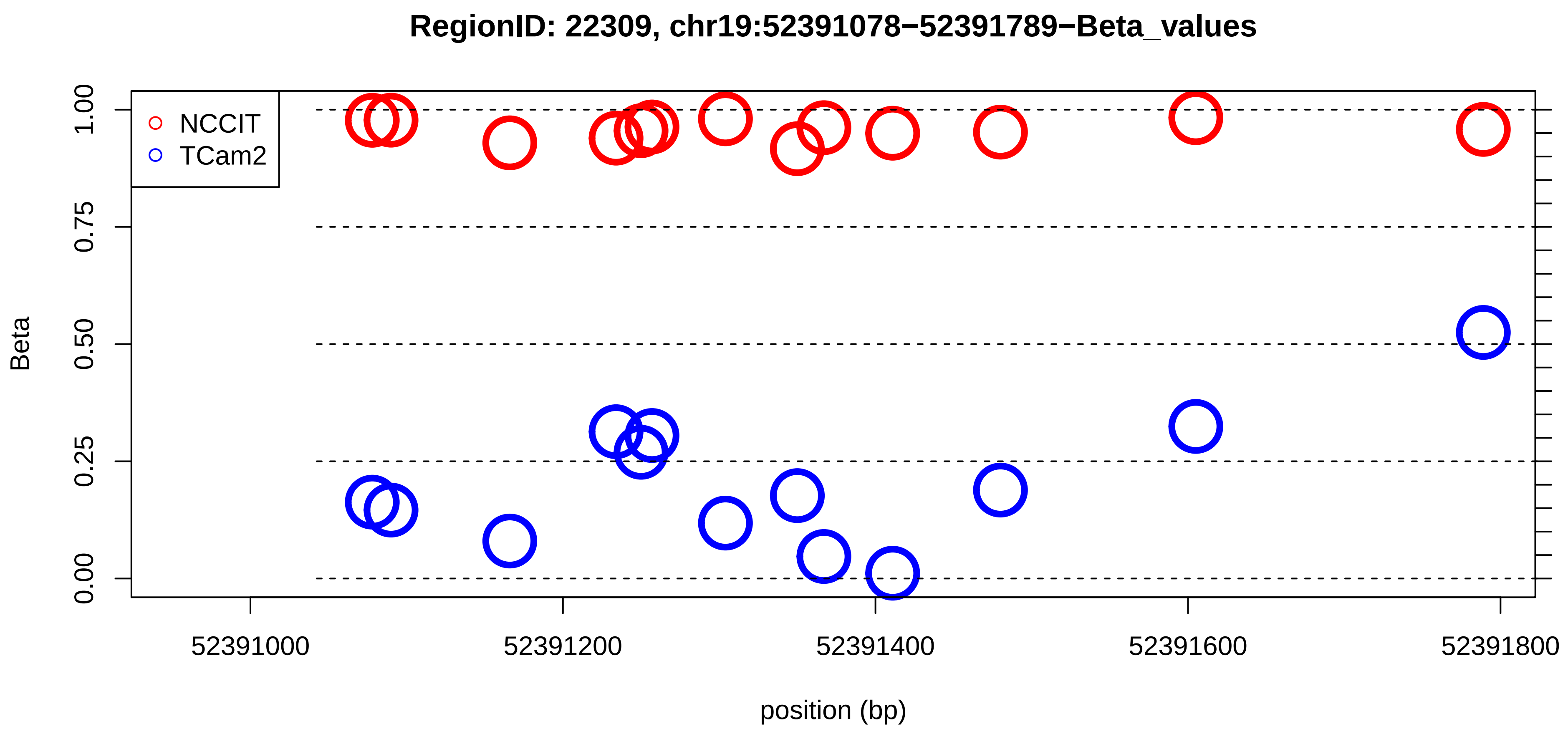
RegionID: 22309, chr19:52391078−52391789−Beta_values
NCCIT
TCam2
Beta
1.00
0.75
0.50
0.25
0.00
52391000
52391200
52391400
52391600
52391800
position (bp)

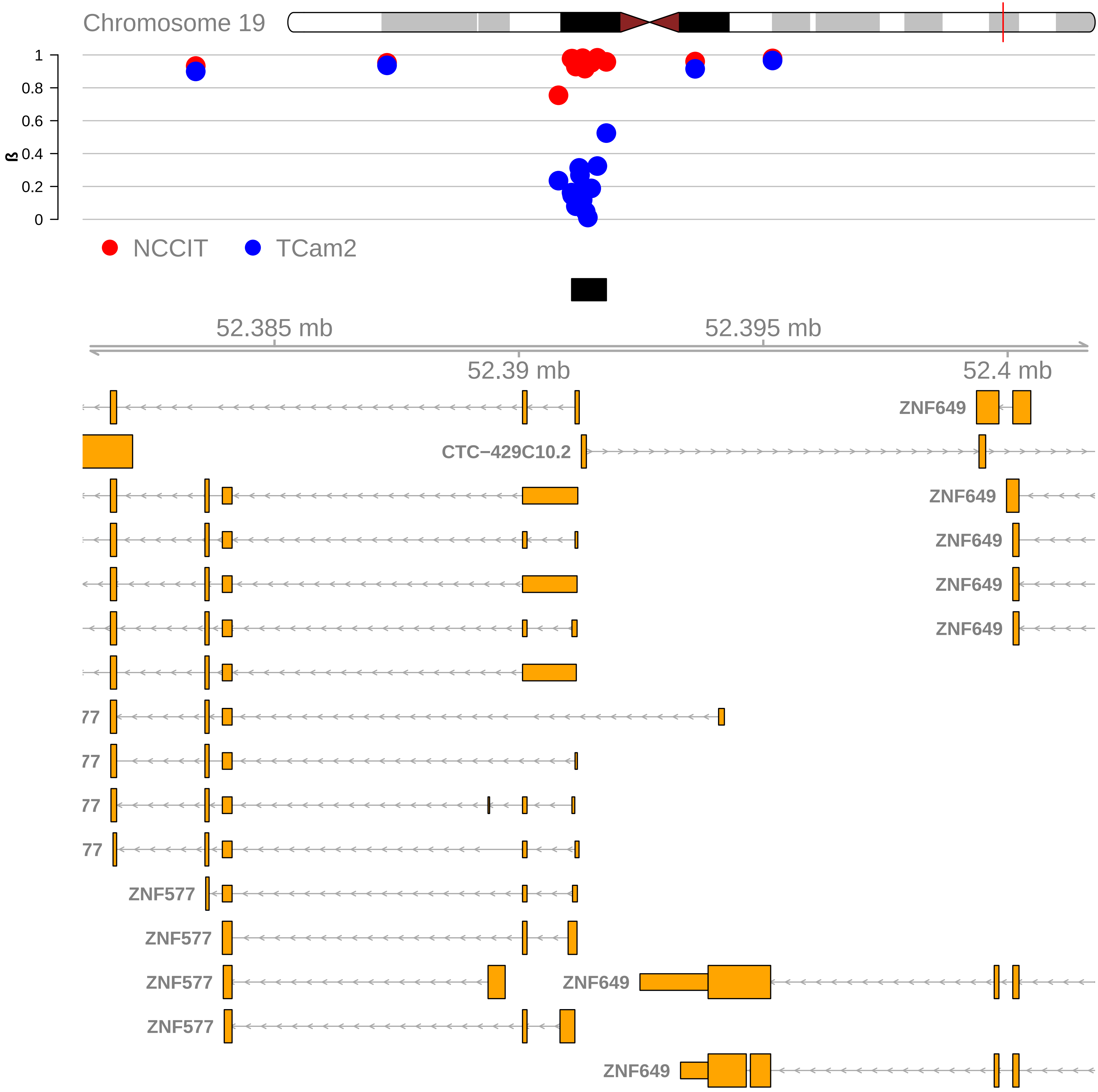

Chromosome 19
ß
1
0.8
0.6
0.4
0.2
0
NCCIT
TCam2
52.385 mb
52.395 mb
52.39 mb
52.4 mb
ZNF649
CTC−429C10.2
ZNF649
ZNF649
ZNF649
ZNF649
77
77
77
77
ZNF577
ZNF577
ZNF577
ZNF649
ZNF577
ZNF649
